# Environment shapes sleep patterns in a wild nocturnal primate

**DOI:** 10.1038/s41598-019-45852-2

**Published:** 2019-07-09

**Authors:** Kathleen D. Reinhardt, Vladyslav V. Vyazovskiy, R. Adriana Hernandez-Aguilar, Muhammad Ali Imron, K. Anne-Isola Nekaris

**Affiliations:** 1 Nocturnal Primate Research Group, Oxford Brookes University, Oxford, United Kingdom; 2 Center for Ecological and Evolutionary Synthesis, University of Oslo, Oslo, Norway; 3 Department of Physiology, Anatomy and Genetics, University of Oxford, Oxford, United Kingdom; 4 Department of Social Psychologyand Quantitative Psychology, Faculty of Psychology, University of Barcelona, Barcelona, Spain; 5 Department of Forest Resources Conservation, Faculty of Forestry, Universitas Gadjah Mada, Yogyakarta, Indonesia

**Keywords:** Behavioural ecology, Sleep, Animal physiology, Ecophysiology, Circadian regulation

## Abstract

Among primates, the suborder Haplorhini is considered to have evolved a consolidated monophasic sleep pattern, with diurnal species requiring a shorter sleep duration than nocturnal species. Only a few primate species have been systematically studied in their natural habitat where environmental variables, including temperature and light, have a major influence on sleep and activity patterns. Here we report the first sleep study on a nocturnal primate performed in the wild. We fitted seven wild Javan slow lorises (*Nycticebus javanicus*) in West Java, Indonesia with accelerometers that collected activity data, and installed climate loggers in each individual’s home range to collect ambient temperature readings (over 321 days in total). All individuals showed a strictly nocturnal pattern of activity and displayed a striking synchronisation of onset and cessation of activity in relation to sunset and sunrise. The longest consolidated rest episodes were typically clustered near the beginning and towards the end of the light period, and this pattern was inversely related to daily fluctuations of the ambient temperature. The striking relationship between daily activity patterns, light levels and temperature suggests a major role of the environment in shaping the daily architecture of waking and sleep. We concluded that well-known phenotypic variability in daily sleep amount and architecture across species may represent an adaptation to changes in the environment. Our data suggest that the consolidated monophasic sleep patterns shaped by environmental pressures observed in slow lorises represent phylogenetic inertia in the evolution of sleep patterns in humans.

## Introduction

Sleep is a fundamental requirement for many animals in maintaining cognitive performance and physiological functions^1–3^. Birds and mammals can display two different sleep patterns: monophasic sleep, when an animal exhibits a single consolidated bout of sleep in one portion of a day; or polyphasic sleep, when an animal displays several short episodes of sleep^2,4,5^. Even in relatively stable laboratory conditions, the amount and characteristics of sleep and waking vary substantially across 24-h. These variations are superimposed with daily fluctuations in environmental variables that have a strong influence on activity patterns. Such fluctuations include risk of predation, ambient temperature, humidity and light^5–10^. The effect of these extrinsic factors on sleep and activity is mediated by their interaction with endogenous regulatory mechanisms, such as the circadian clock^11^. The circadian clock provides a rhythmic output to behaviour and physiology, and is synchronised to light levels, allowing animals to anticipate day and night^8,12^. This synchronisation is due to retinal photoreceptors and their photosensitivity to light cues, commonly referred to as Zeitgeber^8^. Another important intrinsic factor, which has a strong influence on sleep amount and intensity, is preceding sleep-wake architecture. Despite growing literature on sleep and its functions in the last decades, the need for comparative research in natural environments to further our understanding of sleep ecology, physiology and evolution is becoming increasingly important^2,5^.

Numerous laboratory studies suggest that sleep is homeostatically regulated^13^. The intensity of sleep increases as a function of preceding wake duration, with lowest sleep pressure towards the end of a sleep period^14–17^. An important manifestation of sleep homeostasis is the capacity to compensate for the loss of sleep following sleep (or rest) deprivation^3,18^. An animal that experiences a stimulus that arouses them from sleep during regular circadian rest patterns would need to reallocate resting time at another portion of the day^5^. Sleep rebound is regularly observed in laboratory animals, and suggests importance of the restorative function of sleep, and the detriments an animal can experience should amount of time for sleep be compromised^3,18–22^. To our knowledge, no studies on instrumental sleep deprivation have been performed in the wild, where we surmise that the need to compensate for sleep loss would need to be balanced against environmental pressures^10^. One study conducted on wild African elephants (*Loxodonta africana*) found no evidence of sleep rebound in response to prolonged continuous spontaneous wakefulness^23^. Similarly, wild fur seals (*Callorhinus ursinus*) did not display REM sleep rebound when they returned to land after prolonged loss of REM sleep while in the water^24^.

Sleep patterns in several mammalian species have been systematically studied in their natural habitat, but little is known about sleep in wild primates^23,25–29^. Evidence suggests that nocturnal primates display on average longer sleep durations (13–17 h daily) compared to diurnal species that sleep for 8–11 h^27,30^. Captive studies of nocturnal primates (African lesser bushbaby *Galago senegalensis*, greater slow loris *Nycticebus coucang*, Northern owl monkey *Aotus trivirgatus*) display strictly nocturnal activity^10^, but it is unclear whether these patterns extend to the wild^31–34^. We aimed to bridge this knowledge gap on the environmental drivers of sleep in wild animals by conducting the first study on sleep patterns in a wild nocturnal primate, the Javan slow loris (*Nycticebus javanicus*).

We utilised actigraphy as a method to measure behavioural sleep (rest) of *N. javanicus* in the wild using collar-mounted accelerometer devices. Previous studies used activity monitoring in marmosets (*Callithrix jacchus*) to examine the diurnal rest-activity cycle^35^ and this approach was validated against EEG recordings^36^, confirming that actigraphy-defined immobility is a suitable proxy for sleep in primates. We hypothesised that under natural conditions, environmental variables would strongly influence the daily activity patterns and sleep behaviour of *N*. *javanicus*. We predicted that: 1.) *N*. *javanicus* would display diurnal sleep of a shorter duration than observed for nocturnal primates in captivity; 2.) *N*. *javanicus* would perform consolidated monophasic sleep patterns; 3.) Light and/or temperature would mediate their activity patterns; 4.) *N*. *javanicus* would display resting patterns that suggest homeostatic sleep regulation.

## Materials and Methods

All research was conducted in adherence to the ethical practice and guidelines provided by the Association of the Study of Animal Behaviour, as well as the Indonesian Ministry of Science and Technology, RISTEK (1421/FRP/SM/VIII/2015). All research was additionally approved by the University Animal Ethics Sub-committee of Oxford Brookes University in the United Kingdom.

### Study site and subjects

We studied *N*. *javanicus* on Mount Puntang on the Indonesian island of Java, which is part of the Java-Bali Rain Forests ecoregion. This population of slow lorises is found around Cipaganti in Garut Regency (7°16′44.30″S, 107°46′7.80″E, 1200 m asl), ranging between 1,250 and 2,364 m asl. This region is a sub montane environment near the equator, where temperatures vary greater on a daily range (typically between 16–35 °C) than they do annually^37^.

The Javan slow lorises studied here are part of a population of wild individuals routinely monitored and studied *in situ* by an on-going (2012 – present) research project, the Little Fireface Project^38^. Individuals within this population have displayed a broad variation in home range size (2–19 ha^39^). They are routinely fitted with cable-tie radio transmitter VHF collars (Biotrack TW3, Wareham, United Kingdom) secured around their necks for individual identification. Due to the nature of slow loris locomotion (e.g. slow climbing, cantilevering), the placement of collars around the neck has been found most efficient for recording activity of *N*. *javanicus*^38,40^. To attach radio transmitter collars, animals are captured from trees by an experienced Indonesian field assistant using protective gloves. Capturing was done without anaesthesia and at a minimal frequency, to replace radio transmitter batteries (average 12-month battery life) or to retrieve accelerometers (average 3-month battery life).

All collared slow lorises were monitored on a regular rotating basis using Sika receivers and Yagi antennae (Biotrack Ltd., Wareham, United Kingdom) and head torches with red filters (Cluson Engineering Ltd., Hampshire, United Kingdom) between 17:00 and 05:00. Monitoring occurred an average of two nights per month using scan sampling to make sure collars remained properly fitted and individuals were performing natural behaviours^41^.

#### Accelerometer recordings

**Figure 1:**
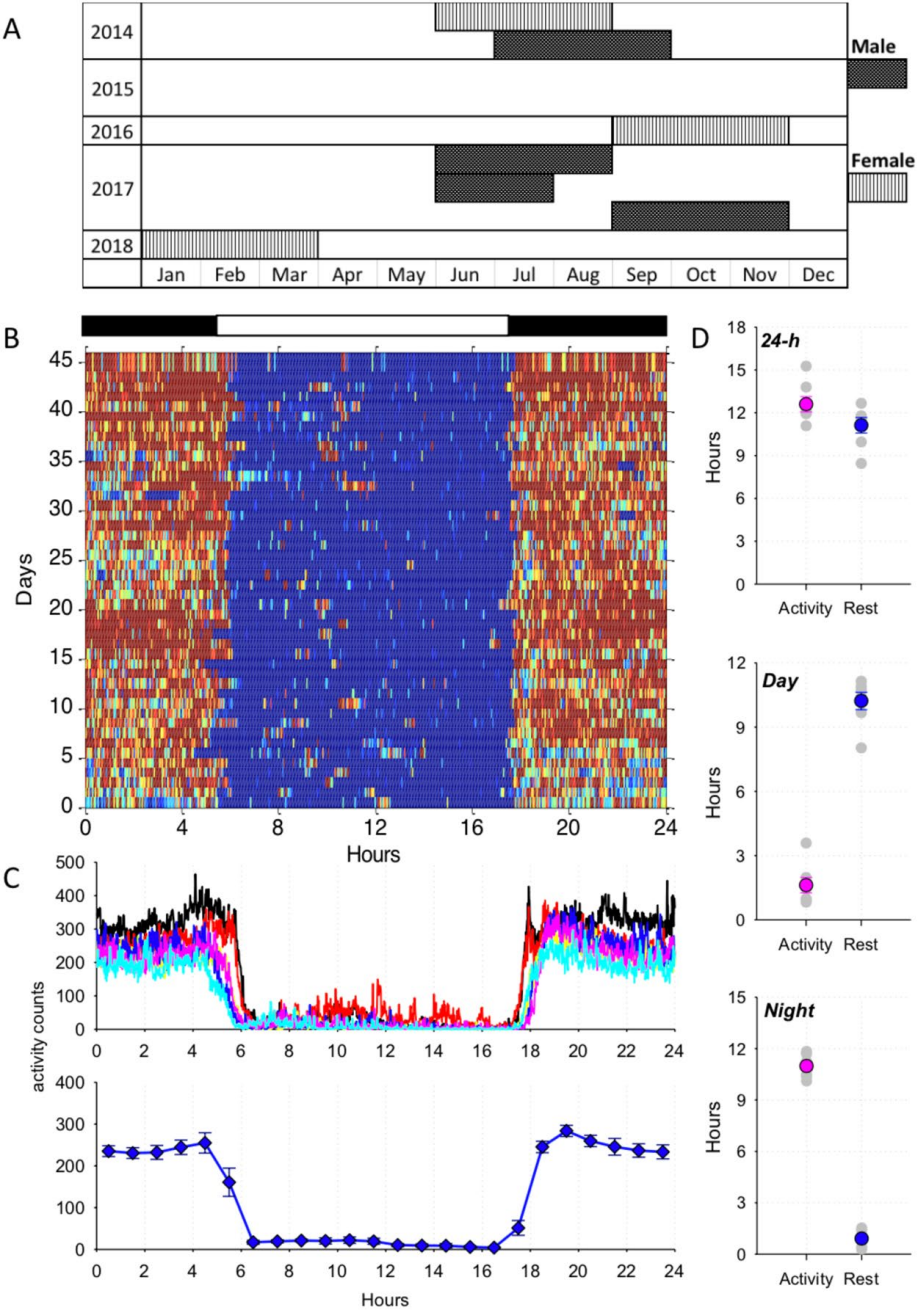
(**A**) Timeline of accelerometer recordings on individual animals (n = 7) shown separately for males and females. Each row represents one individual loris. (**B**) Representative 3-D actogram of the mean activity distribution of one individual loris across 24 h. Activity is represented in a colour gradient, where blue schematically depicts inactivity and red epochs correspond to activity. The bars above depict the night (hours between sunset and sunrise) and day (between sunrise and sunset). (**C**) Top: average activity profile plotted for each individual loris over 24-h; 1-min resolution. Each colour represents a different individual; bottom: average activity (SEM, n = 7) shown in 1-h intervals. (**D**) Proportion of time spent active and inactive (rest) over 24-h, during the day and during the night. Mean values (SEM) are shown as coloured symbols. Individual values are shown in light grey.

To quantify activity and rest of *N*. *javanicus* we equipped electronic accelerometer devices (Actiwatch Mini: CamNtech Ltd., Cambridge, UK) to the VHF radio collars of twelve individuals on a rotating basis between June 2014 and April 2018 (see Fig. 1). Accelerometers were programmed to store full activity counts at 1-min epochs using MotionWare software (CamNtech Ltd., Cambridge, UK). Collar-mounted accelerometers have been found most suitable for wild primates because they do not disrupt their normal behaviours and are most convenient for long-term actigraphy monitoring^35,42^. The use of neck-collars is also best suited for comparative research conducted on arboreal primate species, as this method decreases the risk of animals getting caught in trees or branches^43–47^. The combined weight of radio-collars (19 g) and accelerometers remained well under the recommended five percent of the body weight of study animals, with *N*. *javanicus* body mass averaging at 903 g, and ranging between 850 and 1100 g^41,48^.

### Data processing

**Figure 2:**
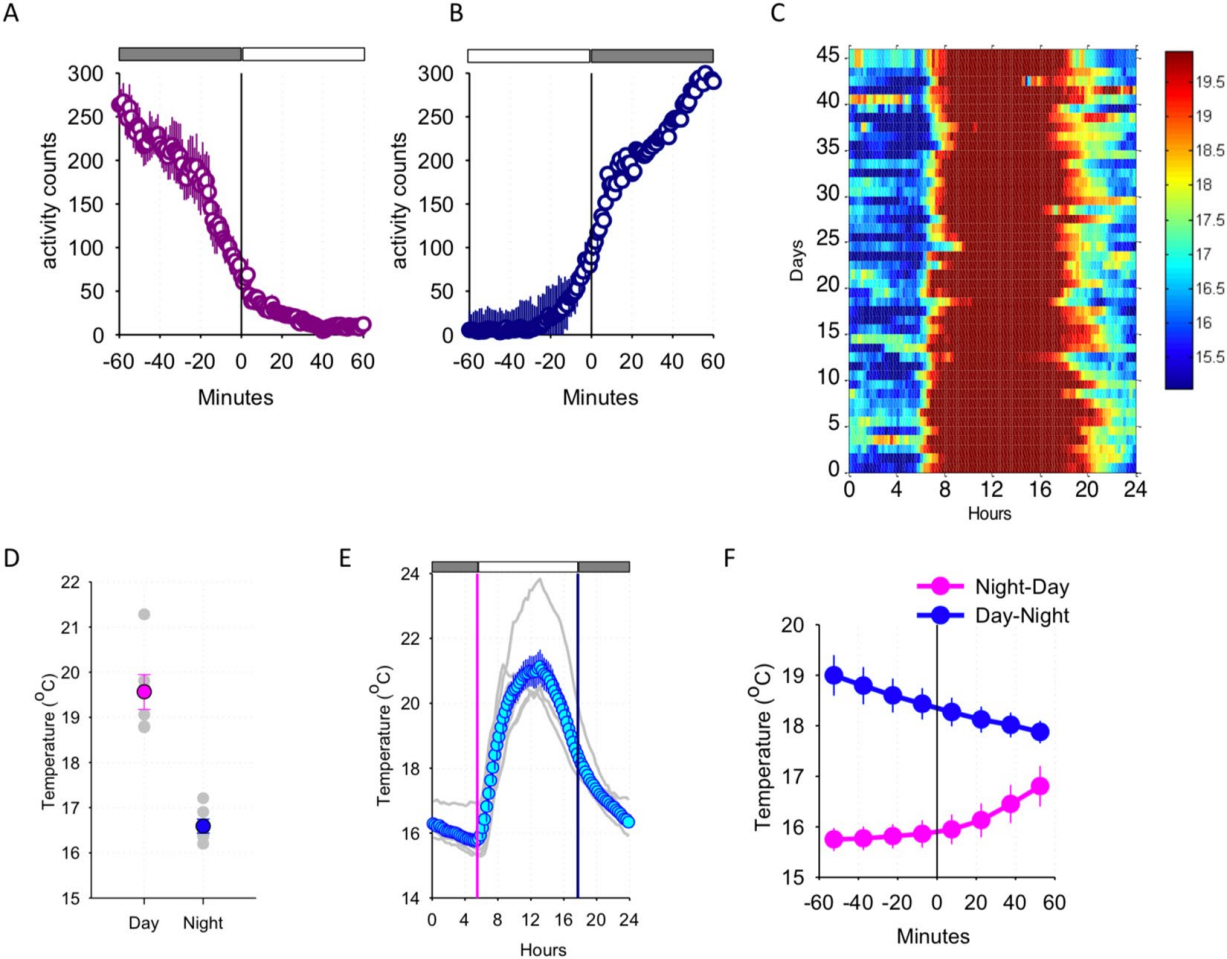
(**A**) The time course of locomotor activity at the transition from night to day (depicted as the bar above the curves). 1-min values of activity (first averaged between days within an individual, prior to calculating means between individual lorises, n = 7, SEM) are plotted for one hour prior to sunrise and one hour after the sunrise. (**B**) Same as A for the day-night transition. (**C**) Representative data of ambient temperature recorded from the home range of one individual loris across the entire recording period. The values are colour-coded according to temperature from warmer (dark red) to cooler (blue), as shown on the scale bar on the right. (**D**) Mean values of ambient temperature during the day and during the night. N = 6 animals, SEM. Individual values are shown in light grey. (**E**) The time course of average ambient temperature recorded from the home ranges of all individual lorises (n = 6, SEM). 15-min values are plotted consecutively from midnight till midnight next day. Grey curves represent ambient temperatures for each individual; blue circles represent the mean value for each 15-min temperature recording across a 24-h period. Vertical lines depict average time of sunrise (magenta) and sunset (dark blue). (**F**) Time course of ambient temperature during the corresponding 2-h intervals as shown in (**A**,**B**). Mean values (n = 6) are plotted in 15-min intervals.

**Figure 3:**
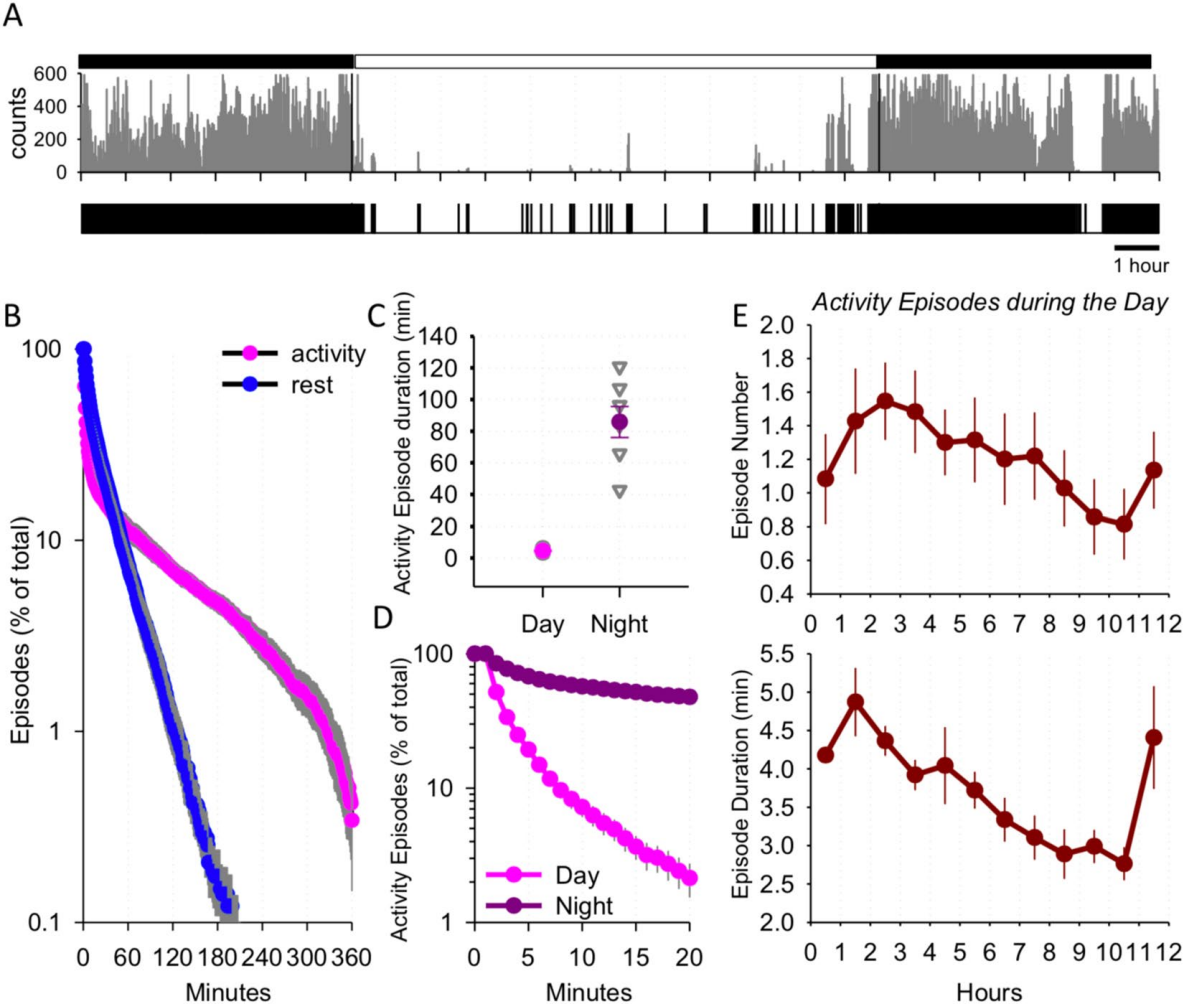
(**A**) Representative profile of activity shown across 24-h starting at midnight. The lower scale is a binary scale of the above measurements, where 0 is representative of complete immobility (no activity counts)and 1 corresponds to 1-min bins with at least one count of activity. Note that most activity occurs during the night, but occasional short activity bouts occur also between sunrise and sunset. (**B**) Distribution of rest episodes (defined as periods with 0 activity) as a function of their duration. Prior to calculating averages between days and individuals, all rest episodes were detected and plotted against their progressively increasing duration and expressed as % of the total number of rest episodes. Mean values, n = 7, SEM. (**C**) Mean duration of activity bouts during the day and night. SEM, n = 7. Individual values are shown as light grey symbols. (**D**) “Survival curves” of activity episodes during the day and night. The proportion of activity episodes is plotted as a function of their progressively increasing duration. Note that only a small fraction of activity episodes during the day “survives” beyond 5–10 min, while most activity episodes during the night are sustained for at least 20-min. Mean values, n = 7, SEM. (**E**) Top: time course of activity episode number during the day. The values are plotted in 1-h intervals from sunrise to sunset. Mean values, n = 7. Bottom: time course of activity episode duration during the day. The values are plotted in 1-h intervals as above.

Raw data (activity scores) were extracted from accelerometer devices using the same software we used for launching the loggers (MotionWare software). Using the raw data, we analysed activity scores using Microsoft Excel and MATLAB version R2017b. While the methodology for activity recording in wild primates may need to be further improved in future studies, our data analysis suggests that the temporal resolution and sensitivity of our approach was optimal to capture animal’s activity across a wide range of movement intensities and speed (see Figs 2 and 3).

Accelerometer devices record movement and locomotion as a complete activity score. One of the main defining characteristics of sleep is immobility, yet an animal may be awake while immobile^49–52^. While simultaneous behavioural observations can be used to extrapolate specific behaviours from activity scores, basic latent behaviours can be extrapolated from accelerometers using unsupervised algorithms for larger datasets or cryptic species^53–55^.

We used actigraphy scores to devise criteria for behavioural sleep/rest^56–59^, defining it as complete immobility (activity score equal to zero). Using survival curve analyses, we plotted the distribution of rest episodes during the night and during the day as a function of their progressively increasing duration, expressed as a percentage of the total number of episodes; we performed this same analysis for episodes of activity to quantify how long episodes with movement were sustained.

**Figure 4:**
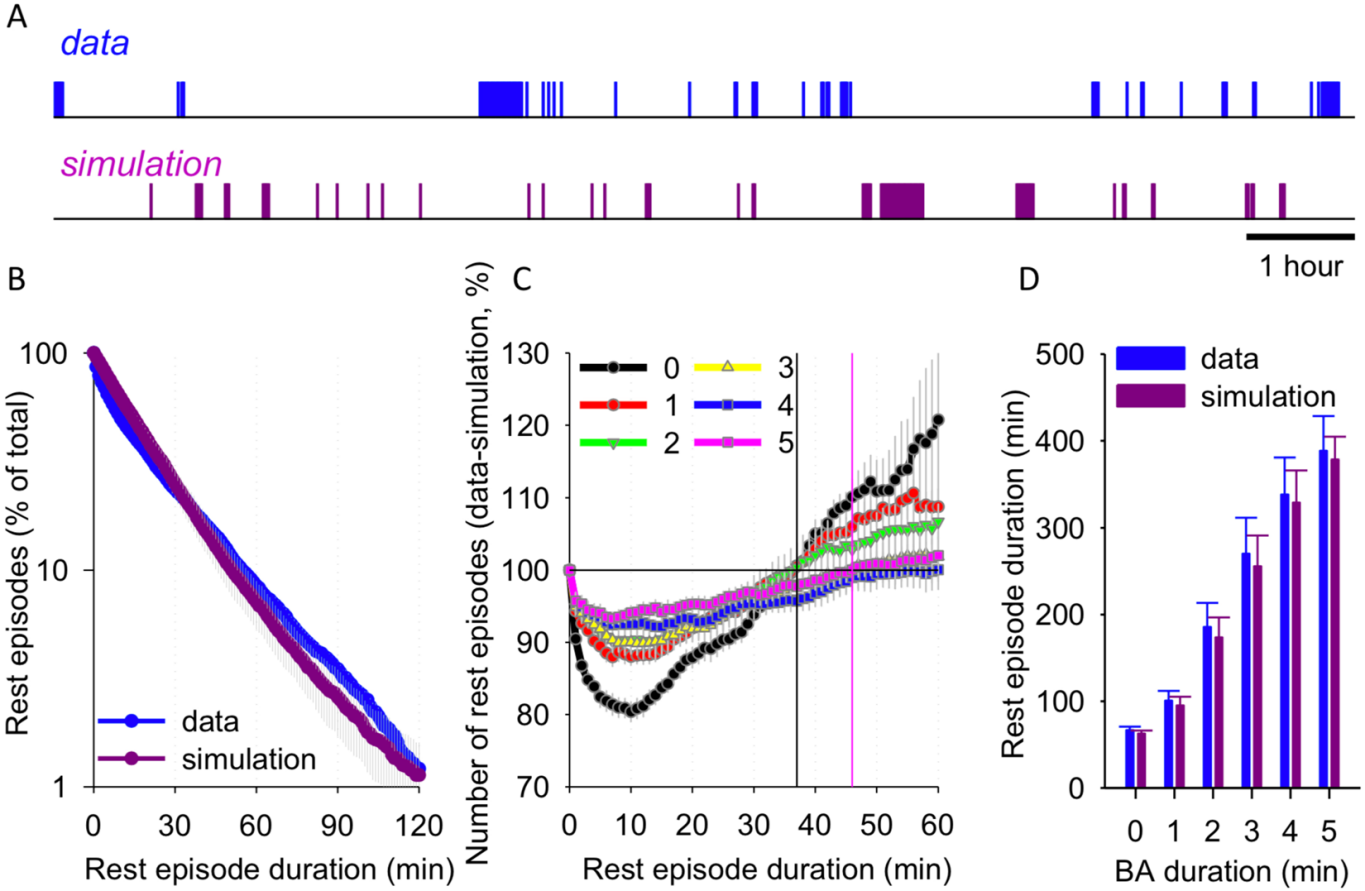
(**A**) Representative profile of activity (where 1-min bins with activity > 0 shown as 1) across one light period between sunrise and sunset. The plot below shows reshuffled activity periods of the same recording, where the number and duration of short activity bouts are retained as above, but their timing of occurrence is randomised. (**B**) Survival analysis of inactivity (rest) episodes for empirical data and simulated activity profiles as above. Note that shorter rest bouts are more likely to occur in the simulated activity time profiles, while longer sustained rest periods occur more frequently in the real data. (**C**) The difference in the distribution of rest episodes derived from data and simulation, calculated as in (**B**). An additional interruption criterion is introduced from 0–5 min. Vertical lines depict minimal rest episode duration where the rest episode duration in data is below simulation for rest episodes where no interruption is allowed (black) and where up to 5-min brief awakenings are permitted (magenta). (**D**) Mean duration of rest episodes derived from empirical and simulated data sets, shown as a function of the interruption criterion. Mean values, n = 7, SEM.

**Figure 5:**
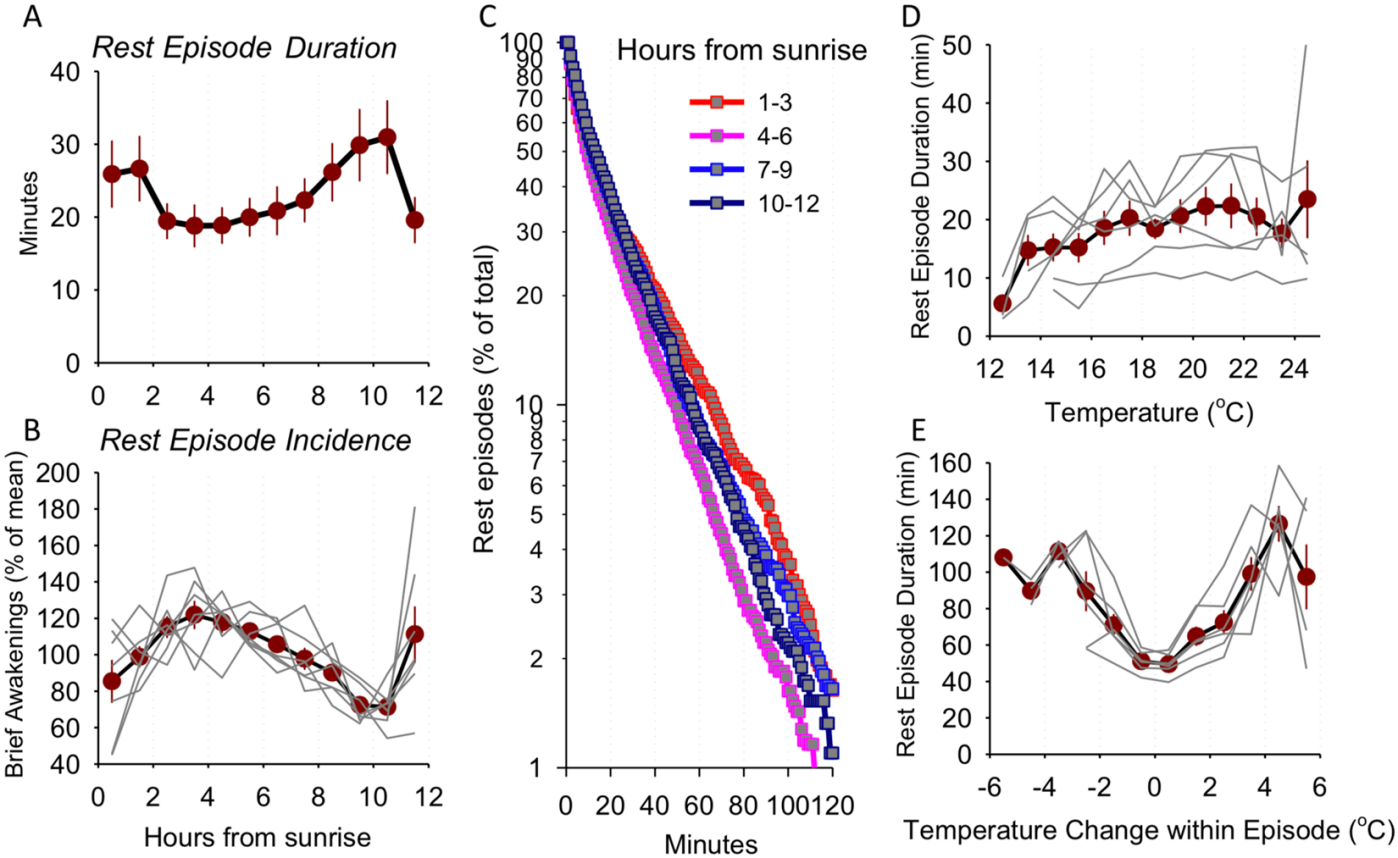
(**A**) Time course of rest episode duration across the light period. Mean values (n = 7, SEM) are shown as dark red symbols. (**B**) Time course of rest episode number across the light period. Mean values (n = 7, SEM) are shown as dark red symbols. The values are expressed as percentage of mean across the entire day. The curves for individual animals are shown in grey lines. (**C**) Survival analysis of inactivity (rest) episodes shown for 3-h intervals across the day. Note that rest episodes “survive” for longer at the beginning of the day, then their duration drops and tends to increase again in the second half of the light period. Mean values, SEM, n = 7. (**D**) The relationship between ambient temperature and rest episode duration during the day. All rest episodes during the day are grouped according to the ambient temperature at the time of their occurrence. Note that at warmer temperatures the rest episodes tend to be longer. (**E**) The relationship between change in ambient temperature within sustained rest episodes and their duration. All rest episodes are grouped according to the magnitude of change in temperature, and the corresponding values are averaged. Note that rest episodes tend to be longer if the temperature is decreasing or increasing, but remain short if the temperature is stable. Mean values, n = 7, SEM.

We refer to brief interruptions of rest lasting 5-min or less as “brief awakenings” (see Figs 4 and 5). Note that since no polysomnography data are available and the criteria are based on locomotor activity only, it cannot be determined whether the animals are awake during the entire duration of a ‘brief awakening’ epoch. To investigate the patterns of occurrence of brief awakenings during the day, we used data simulation approach of the retrieved complete loggers. To this end, we reshuffled brief awakenings from the same accelerometer recording, maintaining their quantity and duration but randomising their occurrence in simulated data.

**Figure 6:**
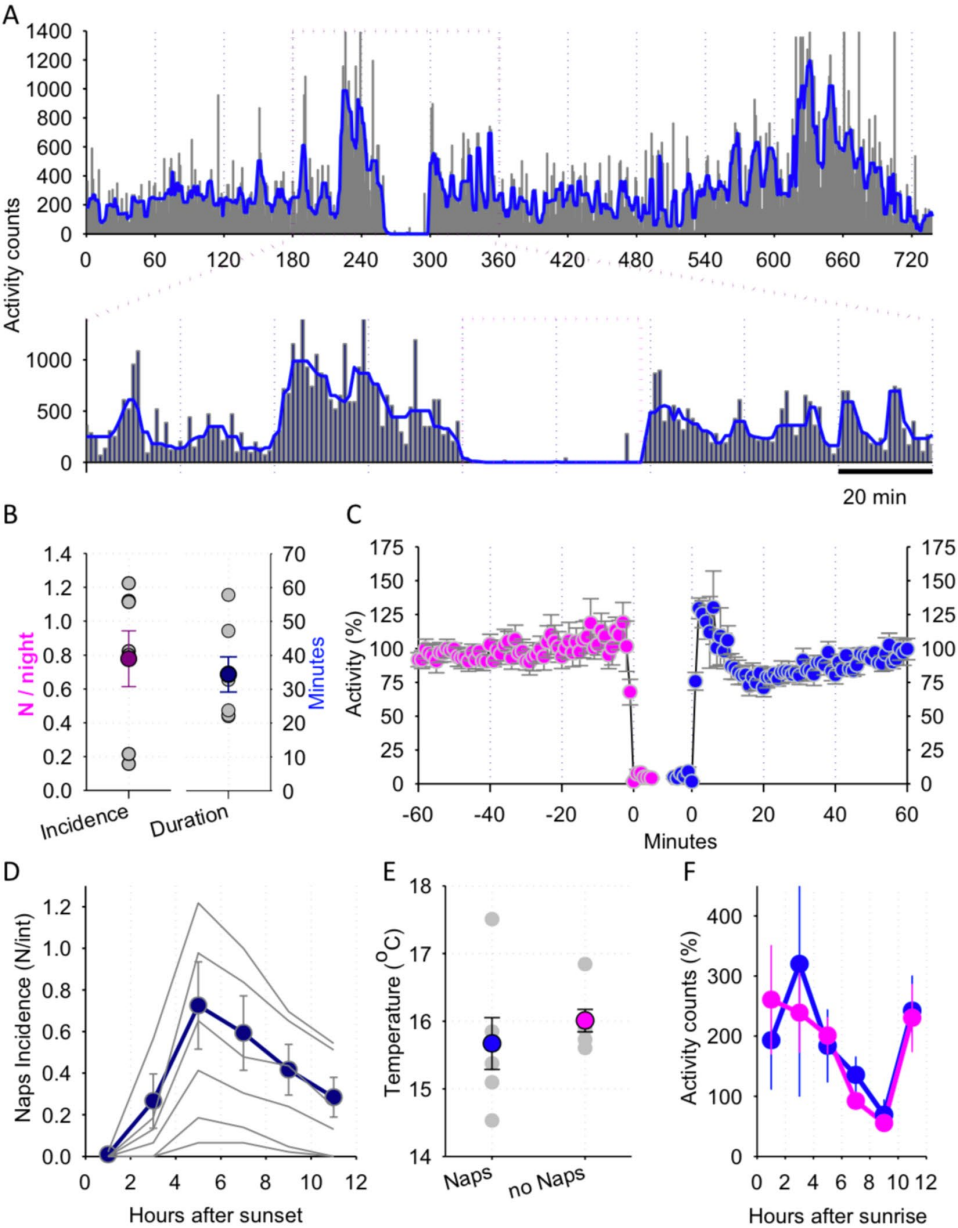
(**A**) Representative profile of activity shown across one night in one individual loris. Note the occurrence of a period of inactivity (nap) approximately 4.5-h after sunset (expanded below). (**B**) Mean values of nap incidence and duration during the night across the entire recording period. Mean values, SEM are shown as coloured symbols and individual animals (n = 7) are shown in grey. (**C**) The time course of activity around the nap. All naps lasting at least 10-min are aligned to their onset and offset and activity is averaged in 1-min bins over 1-h period before and after the nap. Note a surge of activity immediately after the nap. Mean values, n = 7, SEM. (**D**) The time course of nap incidence during the night. All naps lasting at least 10-min were detected across the entire recording period, and grouped according to the timing of their occurrence between sunset and sunrise. Note that the probability of nap occurrence is low during the early hours of the night, but increases progressively towards the middle of the night, and then decreases prior to morning hours. (**E**) The relationship between the occurrence of naps and ambient temperature. For each night across the entire recording period the occurrence of naps was determined and subsequently corresponding mean nightly temperature values were calculated separately for the night with at least one nap, and those nights where no naps occurred. Mean values, SEM are shown as coloured symbols and individual animals are shown in grey. (**F**) Time course of activity during the days following those nights where the animals took naps (blue curve) and those nights where the animals were continuously active. 2-h mean values (n = 7, SEM) of activity are represented as percentage of mean activity during the preceding day.

Naps (periods of consolidated nocturnal inactivity) were defined as periods with zero activity lasting at least 10-min, which were not interrupted by transient epochs of activity longer than 2-min (see Fig. 6).

Our definition of naps equates to an arbitrary minimal duration of 10-min, and is based on extensive visual screening of the data. Generally, we observed an occurrence of consolidated periods of inactivity that were not interrupted by gross movements, if the animal was immobile for at least 10-min. Future studies may provide better criteria for naps when EEG recordings or other approaches to quantify sleep in slow lorises become available.

#### Measuring light environment and ambient temperatures

As light levels can be derived from sunrise and sunset, we gathered all sunrise and sunset times from an online world clock source (Time and Date AS 1995–2018) in the appropriate time zone (GMT +1) at the study site to test for circadian synchronisation. Ambient temperatures were extracted from Hygrochron iButton climate loggers (Maxim/Dallas Semiconductor Corp., USA) that were installed in each slow loris’ home range. Loggers were placed out of direct sunlight on a lower tree branch for the full duration that individuals were fitted with accelerometers, and recorded measurements of ambient temperature at 5-min intervals with an accuracy of ±0.5 °C. Due to water exposure (rain and humidity) one of the retrieved climate loggers did not record data. The temperature measurements from one animal’s home range therefore were not included in the analyses where temperature values were used.

### Quantification and statistical analyses

Data analyses were performed using Microsoft Excel and MATLAB version R2017b. Group data are presented as mean ± SEM, or as individual points when showing representative data from a single animal. Each data representation type is specified in the respective legend for each figure.

We performed non-parametric Wilcoxon signed-rank tests to compare the mean proportion of time individuals spent in activity between the day and night (Fig. 1D), as well as the mean ambient temperature levels (Fig. 2D). We also compared ambient temperatures during bouts of activity/inactivity (Fig. 3C), and during the night with and without naps (Fig. 6E). We performed Repeated Measures ANOVA tests to assess changes in the duration of brief awakenings (Fig. 3E) and the duration of resting bouts (Fig. 5A) across time, between sunrise and sunset.

To test for homeostatic sleep regulation, we first measured the duration of sustained periods of locomotor inactivity as representative for rest consolidation during daytime rest, assuming that least disruptions from rest represent higher sleep intensity.

## Results

Only seven of the twelve retrieved accelerometers had complete data stored. We omitted any loggers with saturated activity scores from our analyses, as these data were likely skewed by water damage, and non-skewed data could not be properly distinguished. Of the retrieved complete loggers, we acquired accelerometer data on four females and three males, of which six were adults and one was a juvenile (female). Retrieved accelerometers contained data for an average of 46.3 ± 0.8 days per logger, contributing to a collective 321 days of data between the individuals (Fig. 1A).

### Light environment drives nocturnal activity and monophasic diurnal rest

Over the duration of this study, the average time (hh:mm ± SD) of sunrise was 05:49 ± 00:12 while sunset occurred at 17:51 ± 00:31. The amount of locomotor activity individuals performed was significantly lower during the day compared to night, where a striking consistency was observed between days with respect to activity offset and onset within and across individual lorises (Fig. 1B: representative individual; Fig. 1B,C: mean values). All animals displayed elevated continuous nocturnal activity pattern with prolonged periods of behavioural rest occurring exclusively during the day (Fig. 1C). The total daily amount of activity (defined as 1-min epochs with at least 1 activity count) and inactivity (defined as 1-min epochs with 0 activity counts) were on average similar (activity: 12.6 ± 0.5, rest: 11.1 ± 0.5 hours; *Z* = 1.521, *p* = 0.128), with periods of inactivity during the dark period being rare, and the reverse pattern displayed during the light period (Fig. 1D; *Z* = 2.3664, *p* = 0.018).

Changes in activity anticipated day-night transitions, where all animals typically displayed an onset and cessation of activity in close proximity to sunset and sunrise. Individuals began transitioning from active to inactive state approximately one hour prior to sunset (Fig. 2A) and transitioned from inactive to active state approximately 20-min prior to sunset (Fig. 2B), suggesting that the change in light levels were likely the key variable affecting the onset of activity and rest.

In addition to daily fluctuations in illumination levels, the ambient temperature also varied between the night and day, raising the possibility that it could also influence daily activity patterns, and trigger the onset of activity and rest. Our recordings revealed that during the day, ambient temperature displayed a mean value of 21.28 ± 0.92 °C, and a mean value of 16.90 ± 0.60 °C at night (Fig. 2D; *Z* = 5.905, *p* < 0.001). It was observed that ambient temperature values showed larger fluctuations during the day, peaking at around midday, while they were relatively stable during the night (Fig. 2C). The daily time course of temperature reached an average minimum around sunrise (15.33 ± 1.85 °C), while mean temperature reached an average maximum at midday (21.27 ± 0.39 °C; Fig. 2E). As ambient temperature transitions between the day and night were modest and typically did not exceed 1 or 2 °C (Fig. 2F), we surmise that transition of activity patterns are more likely driven by either anticipation of day and night or directly by changing levels of light.

### Sleep can be quantified from immobile resting behaviour

Although prolonged bouts of inactivity are likely to represent sleep^60–63^, the possibility remains that some portions of inactivity are merely quiet immobile wakefulness. To characterise the daily architecture of activity in lorises, first we used survival curve analyses as an approach to assess continuity of activity and rest bouts (Fig. 3A). We observed that across 24-h, sustained periods of activity lasted significantly longer than sustained periods of rest (*Z* = −2.366, *p* = 0.016), and complete immobility episodes lasting for longer than approximately 2-h were extremely rare (Fig. 3B,D). This observation is consistent with previous studies in laboratory animals and humans, whose sleep is frequently punctuated with brief awakenings^56–59,64–68^. We next quantified the number and duration of brief awakenings occurring during the day, which revealed that these events occur on average 1–2 times per hour and mostly last <5 min (Fig. 3E; *F* (11, 66) = 6.45, *p* < 0.001).

While the occurrence of brief awakenings is an important feature of physiological sleep, they could also represent movement episodes occurring randomly during resting wakefulness^69^. To determine if daytime occurrences of activity represented brief awakenings from sleep, we tested the likelihood of animals to remain immobile longer than could be expected by chance. To this end, we shuffled the timing of all brief awakenings (with their corresponding durations) randomly across the day to quantify if the same distribution of rest bouts is observed in both the original and simulated datasets (Fig. 4A). The resulting time series suggested that randomly placed brief awakenings resulted in premature termination of prolonged rest bouts than is observed in the empirical data. To quantify these rest bouts, we plotted their distribution as a function of their duration for both the empirical and simulated data. We observed that the simulated dataset was more likely to contain short rest periods that lasted <30 -min (Fig. 4B), indicating that rest bouts detected with actigraphy do not occur randomly between movement episodes, thus representing episodes of consolidated sleep. This effect was attenuated when interruption criteria were introduced (Fig. 4C). The lack of difference between the original and the simulated data sets suggests that the timing of brief arousal occurrence contains important information about rest consolidation, beyond merely rest episode duration (Fig. 4D).

### Rest consolidation, intensity and naps are correlated with temperature changes

The key characteristic of physiological sleep is its homeostatic regulation, which is best represented by the levels of EEG slow-wave activity and the occurrence of consolidated periods of sleep or rest, less frequently interrupted by brief awakenings^69^. Calculating the occurrence of rest episode durations between the sunrise and sunset revealed that rest consolidation varied significantly over the course of the day, with longer rest episodes and lower incidences of brief awakenings at the beginning and towards the end of daytime rest (Fig. 5A–C; *F* (11, 66) = 4.64, *p* = 0). This relationship further suggested that the architecture of rest periods, as detected by actigraphy, is not random, but varies consistently across the day. One possibility is that more consolidated rest reflects increased sleep pressure, which is expected to occur at the beginning of the habitual sleep period, as observed in other species, including primates^70,71^. However, we noted that this dynamic is also similar to the daily fluctuations in ambient temperature, which is at its lowest during the intervals when rest was most consolidated (Fig. 2E). To further assess the relationship between ambient temperature and rest episode characteristics, we clustered all rest episodes based on the average ambient temperature during their occurrence, which revealed a positive association. Specifically, rest duration was longer when ambient temperatures raised above 13 °C (Fig. 5D). Change in temperature within rest episodes also showed a relationship, with shortest episodes corresponding to stable temperature levels, but increasing along the steep upward and downward shifts in temperature (Fig. 5E). This result suggested that the duration of rest bouts may largely be determined by temperature levels and their instantaneous fluctuations, yet in some cases it may be partially uncoupled.

Finally, we noted that although the majority of night time activity periods were uninterrupted, the occurrence of consolidated periods of total or partial inactivity was not uncommon, and was encountered in all animals. While it is unknown whether such episodes of inactivity represent merely wakeful immobility or sleep, we tentatively referred to periods of immobility of ≥10 min as putative “naps”. All animals displayed varying quantities and duration of naps in their regular activity patterns, ranging approximately between 10–60 min, with some individuals napping almost daily and others only occasionally (Fig. 6B). We observed a marked surge of activity after a nap (see individual example shown on Fig. 6A), which gradually decreased towards a lower plateau within approximately 20-min after a nap (Fig. 6C).

Next, we hypothesised that the occurrence of naps during the night may reflect increasing sleep pressure. To this end, we calculated the timing of nap occurrence. This analysis revealed that it was highly unlikely that an individual displayed a nap during the first few hours after sunset, while the probability of napping increased substantially towards the mid-portion of the night (Fig. 6D). The levels of ambient temperature did not show a strong association with the occurrence of naps (Fig. 6E; *Z* = −1.363, *p* = 0.173), suggesting that rather than being driven by changes in the environment it is likely to occur spontaneously. The occurrence of naps during the night was unrelated to the amount of activity during the preceding light phase (Fig. 6F), suggesting that their expression is dictated not only by preceding sleep-wake history, but possibly promoted when environmental conditions are favourable. To further investigate the influence of immediately preceding history of activity, we calculated the relationship between the intensity of movement 1-h prior to the first nap on each night and the corresponding duration of naps. In instances where more than one nap occurred during the night, we used the first nap only to avoid the effects of preceding naps, which sometimes occurred in “clusters”. We found a weak positive association between the two (Pearson’s correlation: *r* = 0.29, *p* = 0.0008), suggesting that nap characteristics may reflect sleep need. We emphasise the limitations when interpreting these results as further evidence for sleep homeostasis, as numerous environmental factors or internal factors may influence nap characteristics, such as body temperature, not accounted for with our approach.

## Discussion

Our study is the first to describe and measure behavioural sleep of a nocturnal primate *Nycticebus javanicus* in their natural habitat. We observed that slow lorises displayed generally similar duration of immobility-defined sleep as diurnal primates, maintaining an average of eleven hours of sleep on a daily basis. The most important novel finding was that environmental variables, such as the levels of light and ambient temperature had a major influence, shaping the overall pattern of activity and rest across 24 -h. Increased consolidation of rest at the beginning of their habitual sleep period may reflect increased sleep ‘intensity’ or direct influence of ambient temperature. Our study therefore raises an intriguing question of how wild animals cope with obtaining sufficient sleep, or compensate for sleep loss incurred during spontaneous or enforced wakefulness while adjusting their sleep pattern to predictable and unpredictable fluctuations in the environmental factors.

### Environmental influences on circadian rhythm

Light environment can synchronise a mammal’s circadian clock, regulating both behaviour and sleep^8^. Circadian rhythms allow an animal to predict regular changes in its environment, such as sunrise and sunset^72^. *Nycticebus javanicus* displayed circadian rhythms in activity and rest that were highly synchronised with the light levels. Anticipating sunrise and sunset suggests that in this species the circadian occurrence of activity and rest is strongly entrained to the periodicity of day and night.

In addition to the levels of light, the daily fluctuations of ambient temperature also played a role in sleep architecture. It has been reported in endotherms (including humans) that sleep and circadian rhythms can be altered when an individual is exposed to temperatures exceeding one’s thermoneutral zone^5,25,73–76^. The combination of seasonal temperature shifts in conjunction with light level changes can result in dramatic shifts in activity patterns, as seen, for example, in the Arabian oryx (*Oryx leucoryx*)^25^. This species of oryx has been observed to prolong their time spent inactive during the winter when temperatures are cooler, in addition to showing an earlier offset of daily activity during the winter. We observed that slow lorises experienced elongated resting bouts during their daytime rest in response to warmer ambient temperatures. The strong effects of temperature on sleep in slow lorises have important implications for the conservation of this species, as remnant populations of *N*. *javanicus* are largely confined to high altitude habitats as a result of agricultural expansion and deforestation (less than 9% of forest remains on the island of Java). Temperature variation increases at higher altitudes^77^, which would likely affect their sleep consolidation. Thus, if higher elevational gradients are the only remaining habitats for wild populations, slow lorises will likely experience lower sleep intensity.

### Prolonged immobile behavioural rest and sleep intensity

Mammals and birds display homeostatic sleep regulation, where prolonged wakefulness is followed by prolonged rest during subsequent sleep^18,21,78^. Similarly, animals display sleep rebound where sleep is significantly disrupted mid-rest or when there is a loss of sleep (sleep deprivation) altogether^17,20,79^. This rebound is acquired at a later period (such as during regular active periods), or extended on to the subsequent sleep. *Nycticebus javanicus* displayed prolonged immobility at the beginning of daytime behavioural rest, and this pattern suggests that animals experience a deeper sleep in the first portion of rest, following a period of activity, consistent with other studies in humans and laboratory animals^59,80^. Researchers have hypothesised that fewer disturbances during sleep increases the intensity of REM (Rapid Eye Movement) and NREM (non-REM) sleep, which is referred to as the “sleep quality hypothesis”^80–83^. Comparative research on baboons and orang-utans found that sleeping structures that promote specific sleep postures increase sleep quality^71^. A previous study on sleeping site selection on the population we studied found *N*. *javanicus* to select bamboo almost exclusively as sleep locations^39^. Lorises display unique vascularisation of their forelimbs (so called *rete mirabile*) that allow them to hold substrates for prolonged periods of time. Thus, in slow lorises the structure and size of bamboo as a sleeping substrate is in accordance with the sleep quality hypothesis, aiding undisturbed sleep^70,71,82^. We also observed that *N*. *javanicus* displayed a higher incidence of naps towards the middle of the night—their usual active period. As there was no indication that nap occurrence was triggered by changes in ambient temperature or light levels, it is possible that these naps reflect increased sleep propensity, indicative of a higher sleep need accumulated from continuous activity. The occurrence of these periods of inactivity during the night was unrelated to the total amount of activity during the preceding day, suggesting that extrinsic factors are an important determinant of napping, above and beyond the intrinsic sleep need. This adaptation also suggests a trade-off of foraging and social behaviour for sleep.

### Accelerometers can provide non-invasive insight on sleep patterns of wild animals

While polysomnography (the recording of EEG, EMG and EOG) is considered the most accurate and efficient method for sleep measurements, it can be particularly difficult to conduct in the wild, especially with primates, leaving gaps in our knowledge on the accurate details of primate sleep in their natural environments^30,71^. The majority of sleep quotas and sleep quality data have been collected from captivity, with a paucity of information from mammals in their natural habitats. Kavanau and Peters^11^ found African and Asian lorisids to display strict nocturnal patterns in captivity, beginning cessation of activity an average of thirty minutes prior to artificial sunrise. We found *N*. *javanicus* to begin cessation of activity approximately one hour prior to sunrise in the wild, displaying earlier anticipation of their light environment than in captivity, suggesting a greater sensitivity to natural light. Therefore, actigraphy represents a very useful tool for ecological research, especially considering highly threatened species for which invasive methods would not be permitted or ethical^84,85^. While we regularly refer to resting bouts as a proxy for sleep, measurements of sleep were derived from accelerometers only, and future studies are necessary to validate immobility in defining sleep in this species. Keeping this limitation in mind, our study suggests that important insights about daily architecture and regulation of wake and sleep can be obtained non-invasively.

### Sleep patterns in wild primates

*Nycticebus javanicus* displayed highly consolidated monophasic sleep patterns. Sleeping at distinct times of day (in this case, dawn until dusk), *N*. *javanicus* conformed to typical patterns of a nocturnal, monophasic mammal^4,5,86^. Most non-human primate species display monophasic sleep patterns with polyphasic sleep patterns typically exhibited by small-bodied rodents and insectivorous mammals^5^. These tendencies are suggested to be both largely due to the metabolic processes unique to sleep^73^, as well as tendencies to be prey to many predators where periodic wakefulness can increase predator avoidance^31^. Researchers have reconstructed polyphasic sleep patterns to be the ancestral trait in mammals^87^, and have suggested that monophasic sleep is a derived trait in the suborder Haplorhini^30^. Our study performed in a nocturnal primate belonging to the basal primate clade, Lorisiformes, suggested that pressures for monophasic sleep also occurred in slow lorises. These results show sleep patterns that likely characterised the earliest primates, challenging the assumption that monophasic sleep arose in the diurnal primates and suggesting that human sleep patterns have a longer evolutionary history than previously suggested. Thus, our findings offer a reassessment of the evolution of sleep architecture in primates.

For monophasic sleepers, the nocturnal species studied so far display longer sleep duration than diurnal species. These studies, however, were conducted in captive settings, and so do not fully reflect sleep behaviour occurring in the environment where predation, food resources, climatic shifts and changing levels of sunlight are considerable restrictions on the amount of time an animal can spend in sleep. Monophasic sleep is suggested to be more efficient because it involves more time in deep sleep, thus requiring a lower total sleep time per day to meet sleep requirements [31; 88]. Our finding that slow lorises perform a relatively shorter total sleep duration in the wild (~11 h daily) compared to other nocturnal primates (ranging from 13–17 h daily in captivity) contradicts the assumption that diurnal primates have evolved to require less total sleep time, as a result of increased sleep intensity^27,30,88^. Overall, *N*. *javanicus* displayed activity patterns that are strikingly synchronised with sunset and sunrise, and rest fragmentation and duration that are correlated to temperature changes. Our results urge more research on the sleep patterns of other wild mammals, particularly primates, in testing hypotheses on sleep traits and how they may be influenced by changes in the natural environment.
